# Measuring client satisfaction and the quality of family planning services: A comparative analysis of public and private health facilities in Tanzania, Kenya and Ghana

**DOI:** 10.1186/1472-6963-11-203

**Published:** 2011-08-24

**Authors:** Paul L Hutchinson, Mai Do, Sohail Agha

**Affiliations:** 1 Department of Global Health Systems and Development, School of Public Health and Tropical Medicine, Tulane University, 1440 Canal Street, Suite 2200-TB46, New Orleans, Louisiana, USA; 2 Population Services International (PSI), 1120 19th Street, NW, Suite 600, Washington DC 20036, USA

## Abstract

**Background:**

Public and private family planning providers face different incentive structures, which may affect overall quality and ultimately the acceptability of family planning for their intended clients. This analysis seeks to quantify differences in the quality of family planning (FP) services at public and private providers in three representative sub-Saharan African countries (Tanzania, Kenya and Ghana), to assess how these quality differentials impact upon FP clients' satisfaction, and to suggest how quality improvements can improve contraceptive continuation rates.

**Methods:**

Indices of technical, structural and process measures of quality are constructed from Service Provision Assessments (SPAs) conducted in Tanzania (2006), Kenya (2004) and Ghana (2002) using direct observation of facility attributes and client-provider interactions. Marginal effects from multivariate regressions controlling for client characteristics and the multi-stage cluster sample design assess the relative importance of different measures of structural and process quality at public and private facilities on client satisfaction.

**Results:**

Private health facilities appear to be of higher (interpersonal) process quality than public facilities but not necessarily higher technical quality in the three countries, though these differentials are considerably larger at lower level facilities (clinics, health centers, dispensaries) than at hospitals. Family planning client satisfaction, however, appears considerably higher at private facilities - both hospitals and clinics - most likely attributable to both process and structural factors such as shorter waiting times and fewer stockouts of methods and supplies.

**Conclusions:**

Because the public sector represents the major source of family planning services in developing countries, governments and Ministries of Health should continue to implement and to encourage incentives, perhaps performance-based, to improve quality at public sector health facilities, as well as to strengthen regulatory and monitoring structures to ensure quality at both public and private facilities. In the meantime, private providers appear to be fulfilling an important gap in the provision of FP services in these countries.

## Background

Numerous studies have examined the effects of family planning quality on the uptake and continuation of family planning methods [1-7]. One principal determinant of uptake and continued utilization of family planning services is overall client satisfaction with those services [8,9]. Studies of contraceptive discontinuation rates, for example, have indicated that - with the exception of the desire to become pregnant - the principal reason for discontinuation is dissatisfaction with the quality of services [10].

Both the public and private sectors supply substantial portions of family planning methods in developing countries, but face different incentives to provide services of high quality and to ensure client satisfaction [11,12]. Public sector health services, for example, are less likely to be motivated by economic incentives (since governments and their health facilities seldom go out of business) and have frequently been characterized by low staff morale, attendance and performance, often related to poor or infrequent pay, at least relative to the private sector [13,14]; poor quality of care and treatment [15]; shortages of workers, medicine, supplies and functioning equipment; and waste and inefficiency [11,15-18].

Motivated to maximize the demand for their services while minimizing their costs, private for-profit facilities generally face greater incentives to be efficient and client-friendly providers of health care. Even so, they have been shown to be of varying quality, often due to the inability of government regulatory bodies to adequately monitor and enforce standards [19-22]. Private providers may also take advantage of informational asymmetries to sell unnecessary - or poor quality services - to unsuspecting consumers [21]. Non-governmental facilities, often not-for-profit and affiliated with religious, faith-based organizations, have been touted as being likelier to provide higher quality services because of their social mission, but evidence to support this has been mixed [13,18].

As calls for privatization and performance-based incentive schemes have become an increasing part of the dialogue surrounding health systems strengthening in developing countries [11,12,23-25], the need for evidence-based assessments of quality differentials between public and private providers has also increased. To date, only a handful of studies have examined differences in the quality of family planning services provided by the public and private sectors [13], and even fewer have sought to link those quality differentials to measures of client satisfaction [26]. As a result, little is known about how moves towards greater private sector provision of family planning will impact upon client satisfaction, contraceptive use, and ultimately fertility.

This study examines differences in technical, structural and process measures of quality between public and private health facilities, both in hospitals and primary care facilities, in three countries - Kenya, Tanzania and Ghana. These countries were chosen principally because of the availability of detailed information on random samples of family planning suppliers via Service Provision Assessments and because the private health sector varies in importance as a provider of family planning across the three countries. Data from Demographic and Health Surveys indicate that the percentage of women receiving contraceptive supplies from private family planning providers ranges from 12.7% in Tanzania (private medical 5.0%; religious/voluntary 7.7%) [27], to 30.5% in Kenya (24.2% private medical; 6.3% mission hospital/clinic) [28], and to 53.7% in Ghana [29]. Further, the family planning situation in these countries is fairly typical for Sub-Saharan Africa. For the region as a whole, the contraceptive prevalence rate is 20.9 percent of women aged 15 to 49 years [30], close to the rates observed in Tanzania (26.4%) [27] and Ghana (23.5%) [29]. Only Kenya has a contraceptive prevalence rate that significantly exceeds this average (45.5%) [28].

This study links measures of FP quality to measures of client satisfaction at each type of public and private family planning provider. Our hypothesis is that higher levels of quality - particularly indicators that measure clients' perceptions of client-provider interactions - will yield higher levels of client satisfaction. In turn, higher rates of client satisfaction have been shown to yield higher family planning adoption and continuation rates [10], though such outcomes are not the focus of this study. Importantly, this study will also assess which specific measures of family planning service quality achieve the largest incremental gains in client satisfaction.

The next section describes the data, the quality measures, and analytical methods utilized in this study. Following that are discussions of the bivariate and multivariate analyses. The last section summarizes the results and discusses some policy recommendations.

## Methods

Service Provision Assessments, developed by ORC Macro [31], are facility-based surveys intended to provide a comprehensive picture of the quality and availability of a basic health services, including those for maternal, child and reproductive health, in a given country. They are intended to be nationally representative of the supply environment, and provide a gauge of how capable existing services are to meet the needs of a country's population. The principal advantages of the SPAs are that they are standardized across countries, thereby allowing direct comparisons in assessments of family planning service availability and quality, and of sufficient sample size to generate sub-national level or facility-type estimates. Further, data collectors, recruited from among nurses and other health professionals trained in survey implementation and interviewing, possess the requisite technical skills to assess the quality and procedural correctness of provider-client interactions.

This study makes use of Service Provision Assessments (SPAs) conducted in Ghana [29], Tanzania [31] and Kenya [32]. In Tanzania, the SPA was led by the National Bureau of Statistics in collaboration with the Ministry of Health and Social Welfare (MoHSW - Mainland and Zanzibar) and the Office of the Chief Government Statistician, Zanzibar. In Ghana, the SPA was carried out by the Ghana Statistical Service (GSS) with assistance and support from the Health Research Unit (HRU), the Ministry of Health (MOH), the Ghana Registered Midwives Association (GRMA), the Planned Parenthood Association of Ghana (PPAG), and the National Population Council (NPC). Finally, the Kenya SPA was undertaken by the National Coordinating Agency for Population and Development (NCAPD), the Ministry of Health (MOH), and the Central Bureau of Statistics. In each country, ORC Macro provided technical assistance.

### Instruments

To provide a broad and detailed picture of the quality and availability of health services and perceptions of quality, the SPAs consist of four standardized data collection components. As noted above, all survey instruments and tools were previously fielded in a number of countries, though country-specific pre-testing was undertaken in each country to ensure that questions were appropriate to local circumstances:

(1) The *Facility Inventory Questionnaire *was used to obtain information on staffing, training, infrastructure, medicines, supplies, and services offered. The focus was on ascertaining the functional ability of facilities to provide services of acceptable standards.

(2) A *provider interview *collected information from samples of health care workers - in particular those who actually provide client services - in order to determine qualifications, experience and perceptions of the service delivery environment.

(3) *Observations of family planning *services were conducted to assess providers' adherence to accepted standards of quality and service delivery.

(4) *Exit interviews *were conducted with clients who received family planning services to determine the clients' experience of the client-provider interaction, recollection of instructions and FP related information, and perceptions of the service delivery environment.

### Sampling

#### Facilities

In each of the countries, health facilities (Table 1) were chosen at random from among the population of public, private, and faith-based facilities that offered services for maternal, child, and reproductive health. Sample sizes were determined based on funding, logistical considerations and minimum sample sizes required when regional estimates were desired. Following similar analyses examining differences in coverage by public and private providers [11], facilities were stratified by operating authority (public vs. private) and by facility type (hospital and other) and a systematic sample was drawn after a random start. Private facilities were defined as those that were either for-profit providers or nongovernmental organizations using market-based approaches to service delivery. In some cases, over-sampling was done to permit analysis by region and facility type, and weights were created to adjust for unequal probabilities of selection.

**Table 1:** Sample of Health Facilities by Country

	Ghana	Kenya	Tanzania
Number of facilities nationwide providing all services	1,444	4,742	5,663
Number selected for survey	428	440	611
Number offering FP services	386	323	482

The final sample of health facilities used in this study - restricted to those which offer family planning services - included 386 in Ghana, 323 in Kenya and 482 in Tanzania (Table 2). In each country, the majority of the health facilities were publicly operated. The weighted sample of hospitals made up 10% of facilities in Ghana, 7% of facilities in Kenya and 4% of facilities in Tanzania. Similarly, private sector providers made up 35% of facilities in Ghana and Kenya and 17% of facilities in Tanzania (Table 2).

**Table 2:** Sample Distribution of Facilities, Provider Interviews, and Client Exit Interviews

		Ghana			Kenya			Tanzania	
	Pct. Distribution (weighted)	Weighted	Unweighted	Pct. Distribution (weighted)	Weighted	Unweighted	Pct. Distribution (weighted)	Weighted	Unweighted
**Facilities**									
Public									
Hospital	6.6	42	42	3.7	12	87	2.2	11	87
Health centers, clinics, dispensaries	58.0	216	185	61.2	198	72	80.4	388	315
Private									
Hospital	3.3	12	12	3.6	12	60	2.0	9.5	24
Health centers, clinics, dispensaries	32.1	116	147	31.5	102	104	15.4	74	56
Total	100.0	386	386	100.0	323	323	100.0	482	482

**Provider Interviews**									
Public									
Hospital	31.1	262	140	26.0	223	310	9.1	113	393
Health centers, clinics, dispensaries	44.6	376	390	40.2	345	161	70.3	874	624
Private									
Hospital	5.6	47	40	13.1	113	192	6.5	81	109
Health centers, clinics, dispensaries	18.7	157	275	20.7	178	197	14.1	175	118
Total	100.0	842	845	100.0	859	860	100.0	1244	1244

**Exit Interviews**									
Public									
Hospital	19.8	121	172	8.9	56	346	6.6	66	411
Health centers, clinics, dispensaries	53.7	328	242	66.7	419	130	83.2	836	493
Private									
Hospital	3.1	19	32	2.9	18	67	2.7	27	58
Health centers, clinics, dispensaries	23.5	143	165	21.5	135	85	7.5	76	43
Total	100.0	611	611	100.0	628	628	100.0	1005	1005

#### Providers

In all three countries, a sample of health care workers/providers was selected from those who were present in the facility on the day of the survey and who provided services in the four areas (child health, family planning, maternal health, and sexually transmitted infections/HIV/AIDS) assessed by the SPA. If a facility had fewer than 8 health care workers, all who were present on that day were interviewed. In facilities with more than 8 providers, at least one provider from each service was interviewed to obtain a minimum of 8 providers. The samples of providers of family planning included 845 providers in Ghana, 859 providers in Kenya and 1,244 providers in Tanzania (Table 2).

### Training and Data Collection

In each country, data collectors were recruited from nurses, clinical officers or social scientists with prior experience in survey implementation and interviewing. Data collectors spent approximately three weeks in training, which included classroom lectures, practical on-site experience in health facilities, and role-playing for observations and exit interviews.

Fieldwork lasted several months, and was undertaken by 13-17 teams of interviewers, generally consisting of one team leader and 2-4 interviewers. In small facilities, data collection took approximately one day, but larger facilities required several days. If a particular service for observation was not offered on the day of a visit, interview teams returned on a day when it was being offered. Interviews with providers of family planning were undertaken with those most knowledgeable of those services at a facility. Informed consent was also obtained from the providers and the facility in-charge.

### Observations and Exit Interviews

Observations were conducted of clients who came for maternal, child, reproductive health or sexually transmitted infection (STI) services. This sample was opportunistic because it was not possible to know how many eligible clients would come to the facility on the day of the observation. Following the observation of client-provider interaction, exit interviews were conducted to determine client satisfaction with services provided. Information on refusal rates for the exit interviews was not available from the published sources or from the data. In total, 611 interviews with family planning clients were conducted in Ghana, 628 interviews in Kenya, and 1,005 interviews in Tanzania (Table 2).

### Operational definitions of quality of care and client satisfaction

Over the years, researchers have developed numerous systems and indicators for measuring the quality of family planning services [6,8,9,33,34]. It is now well-understood that the quality of health services is more than just bricks and mortar availability of infrastructure, supplies or equipment. In this paper, we follow the structure outlined by Donabedian [35], focusing on several categories of quality measures which we describe in detail in Table 3. These include structural, interpersonal and technical attributes of service quality.

**Table 3:** Attributes and Indicators Used for the Assessment of Quality of Care in This Study

	Definition of indicators
**STRUCTURE**	
Infrastructure & equipment	
*Physical infrastructure *	Number of amenities available at facility: electricity, water, working toilet, telephone, waiting area for clients (out of 5)
*Examination room equipment*	Number of following items present: table and stool for gynecological exam, source of light, speculum, soap, single-use towel, water for hand-washing, clean gloves, decontamination solution, sharps box, privacy in exam room (out of 10)
Management	
*Review of management*	Whether there is a system for reviewing management/administrative issues
*System to collect client opinion*	Whether there is a system to obtain clients' opinions regarding services
*Quality assurance program*	Whether the facility has a routine program for quality monitoring
*Supervision*	Whether the last supervisory visit to the facility was in the last 6 months
*Stock inventory, organization, and quality*	Number of following items present at facility: inventory for contraceptive supplies, stock organized by expiry date, contraceptives protected from water, sun, and pests
	
Availability of services	
*Number of days services provided*	Number of days per week that FP services are provided
*Availability of provider *	Whether a trained provider is always available at the facility
*FP methods offered*	Number of methods offered: combined oral pill, progesterone only pill, IUD, 2 or 3 month injectable, 1 month injectable, Norplant, male condom, female condom, spermicide, diaphragm, emergency contraception, counseling about natural methods, tubectomy, vasectory (out of 14)
*Other reproductive health services offered*	Number of RH services besides FP offered: STI services, immunization, antenatal care, postnatal care, postabortion care, and delivery (out of 6)
	
Counseling	
*Guidelines*	Number of guidelines or protocols for counseling at the facility (out of 5)
*Visual aids*	Number of visual aids for demonstrating use of FP methods at facility (out of 9)
*Privacy*	Whether facility has private room for FP counseling
*Individual client card*	Whether there is an individual client card/record for FP
*FP experience of providers*	Number of years of experience of providers in providing FP services
*Providers trained in FP*	Number of providers who received any in-service training in FP in last 5 years
	
**PROCESS**	
Interpersonal	
*Waiting time*	Number of minutes client had to wait before being examined by a provider
*Privacy ensured*	Whether provider ensured visual and auditory privacy during examination
*Client concerns noted*	Whether provider asked client about concerns with methods or with currently used method
*Confidentiality assured*	Whether provider assured client of confidentiality
*Method use explained *	Whether provider explained to the client how to use the method
*Injectable prescription*	Whether provider prescribed an injectable to the client
	
Technical	
*Reproductive history *	Provider asked the client about the following: age, number of living children, last delivery date, history of complications, pregnancy status, desire for more children, desired timing of birth of next child, breastfeeding status, regularity of menstrual cycle (out of 9)
*Physical examination*	Provider took/asked about the following during the physical exam: blood pressure, weight, asked about smoking, asked about STI symptoms, asked about chronic illness (out of 5)
*Injectable procedure *	Provider did the following when giving FP injection: checked client card, wash hands with soap before giving injection, use single-use towel for drying, use newly sterilized needle, stir bottle before drawing dose, clean and air-dry injection site before injection, draw back plunger before injection, allow dose to self-disperse instead of massaging, dispose of needle in puncture resistant container (out of 9)
*Duration of consultation*	Number of minutes provider spent on the consultation
	
**OUTCOME**	
*Client satisfaction*	Clients reported that they had no problem with ALL of the following: waiting time, ability to discuss concerns with provider, amount of explanation given, quality of examination and treatment provided, visual privacy during examination, auditory privacy during examination, availability of medicines at facility, hours of service provision, cleanliness of facility, staff treatment of client

Structural attributes provide an assessment of the overall capacity of health facilities to provide health services. At a bare minimum, the provision family planning services requires at least some minimal level of infrastructure. Specifically, structural attributes of quality were assessed by physical infrastructure, examination equipment, management systems, availability of services and the counseling environment. The Facility Inventory Questionnaire provided the source of data for this component.

Interpersonal and technical aspects of process attributes were considered separately. Interpersonal aspects of quality included maintenance of privacy, confidentiality and provider's handling of client concerns. Prescription of an injectable method by the provider was used as a measure of provider responsiveness to client needs, since the demand for injectables was extremely high among clients who visited these facilities. Technical aspects included elements such as taking a reproductive history, conducting a physical examination and a provider's observation of the correct procedure for administering the injectable contraceptive. The duration of consultation was used as a measure of the technical quality of care. Data for this component came from direct observation of client-provider interactions.

Client satisfaction was measured using clients' responses to questions about service quality, rated as both an index and a discrete measure of problems encountered during the FP visit (none versus any). Specifically, respondents to the exit interviews were asked to report on up to twelve facets of their perceptions of the quality of the visit (Table 4). Rather than examine each of these facets individually, we aggregated them into an index using the *polychoricpca *principal components command for discrete variables using the *Stata 10.1 *statistical software program [36]. While many methods exist for the construction of indexes, this method estimates the polychoric and polyserial correlations amongst the included variables and then performs principal component analysis on the resulting correlation matrix. The first principal component was used as the index for client satisfaction. Alternatively, a discrete measure of client satisfaction was constructed with a value of 1 given for respondents who reported "no problem" with all of the 12 aspects of quality and a value of 0 given for respondents who reported "large" or "small" problem with any of the twelve aspects.

**Table 4:** Measures of Client Satisfaction

Clients were told, "Now I am going to ask you some questions about some common problems clients have at health facilities. As I mention each one, please tell me whether any of these were problems for you today, and if so, whether they were large or small problems for you."
• Time you waited
• Ability to discuss problems or concerns about your health with the provider
• Amount of explanation you received about any problem or method of FP
• Quality of the examination and treatment provided
• Privacy from having others see the examination
• Privacy from having others hear your consultation discussion
• Availability of medicines or methods at this facility
• Hours of service at this facility
• Number of days services are available to you
• Cleanliness of the facility
• How the staff treated you
• Cost for services or treatment
• Any problem you had today that I did not mention

### Data analysis

At the bivariate level, differences in quality of care between private and public sector facilities were assessed. The unit of analysis was the facility level. Because hospitals tend to be larger and offer a wider range of services than clinics, the analysis was stratified into hospitals and all other facilities (clinics, health centers, dispensaries, maternity units and stand-alone VCT centers). T-tests were conducted for continuous variables and chi-squared tests of independence were conducted for categorical variables. To examine the magnitude of the relationship between quality measures and client satisfaction, multiple regression analyses were employed. For the binary satisfaction outcome (i.e., reporting of no problems), a probit model was specified and estimated by maximum likelihood. For the continuous index of satisfaction (e.g. the score of the first principal component of the "problem" index), linear regression was used. In both cases, because clients and providers were nested within facilities, Huber-White standard errors were used to control for the non-independence of client observations clustered at the facility level.

## Results

### Differences in quality of care: bivariate analysis

Table 5 Table 6 and Table 7 compare mean values of indicators representing structural and process attributes of quality by operating authority (private vs. public sector) stratified by facility type for each of the countries. Overall, quality varied more considerably at lower level facilities than at hospitals, and lower level public facilities appeared to be of a slightly lower quality on average than similar-sized private facilities. Fewer differences were detected between public and private hospitals.

**Table 5:** Differences in Attributes of Quality (bivariate analysis) - Tanzania

	Hospitals			Health Centers, Clinics & Other Facilities		
	**Mean** **Value**		**P**	**Mean** **Value**		**P**

	**Public** **(n = 87)**	**NGO** **(n = 24)**		**Public** **(n = 315)**	**NGO** **(n = 56)**	

**BASIC**						
Catchment area population	226,392	106,242	0.204	8,590	7,255	0.401
**STRUCTURE**						
Infrastructure and equipment						
*Physical infrastructure(# of amenities)*	3.72	3.85	0.308	2.51	3.65	0.000
*Examination room (# of items present)*	6.93	6.48	0.227	6.53	7.14	0.022
Management						
*System for review of management (%)*	100.0	89.1	0.056	79.2	85.9	0.440
*System for collecting client opinion (%)*	95.9	89.6	0.928	82.1	39.7	0.000
*Routine quality assurance program (%)*	92.6	86.5	0.211	45.6	40.5	0.586
*Last supervisory visit within 6 mths (%)*						
*Facility has stock inventory and stock is organized and protected (out of 3)*	79.8	60.1	0.004	64.0	44.6	0.001
Availability of services						
*Number of days FP services provided*	5.0	5.2	0.000	4.9	4.6	0.000
*Trained provider always present (%)*	96.9	89.6	0.867	53.4	72.8	0.003
*# of FP methods offered (out of 14)*	6.8	6.1	0.029	4.5	4.0	0.004
*# of other reproductive health services offered (out of 6)*	4.8	4.8	0.270	4.7	4.0	0.000
Counseling						
*# of protocols on FP counseling(out of 5)*	1.5	1.0	0.004	1.2	0.8	0.007
*# of visual aids for demonstrating use of FP (out of 9)*	4.3	2.1	0.000	2.9	2.3	0.011
*Facility has private room for FP counseling (%)*	81.0	64.6	0.650	80.9	71.8	0.089
*Whether there is an individual client card for FP (%)*	97.8	82.3	0.000	81.6	60.1	0.000
**PROCESS**						
*Waiting time*^1 ^*(minutes)*	81.2	81.4	0.988	69.5	25.4	0.000
Interpersonal						
*Privacy ensured during examination (%)*	91.9	100.0	0.025	79.4	74.4	0.644
*Asked clients about concerns with methods or currently used method (%)*	84.1	84.0	0.352	75.0	81.8	0.149
*Confidentiality assured (%)*	77.3	88.5	0.733	58.5	66.5	0.251
*Provider explained method use (%)*	87.4	98.2	0.104	86.2	76.9	0.173
*Provider prescribed injectable (%)*	60.3	52.7	0.492	58.5	49.9	0.398
Technical						
*Reproductive history (out of 11)*	2.9	2.8	0.927	2.2	2.3	0.850
*Physical examination (out of 5)*	2.7	2.8	0.180	2.0	2.6	0.003
*Injectable procedure*^2 ^*(out of 9)*	3.5	3.6	0.699	3.0	3.1	0.701
*Duration of consultation (minutes)*	16.7	16.5	0.887	13.0	13.0	0.986

**Table 6:** Differences in Attributes of Quality (bivariate analysis) - Kenya

	Hospitals			Health Centers, Clinics & Other Facilities		
	**Mean** **Value**		**P**	**Mean** **Value**		**P**

	**Public** **(n = 87)**	**NGO** **(n = 60)**		**Public** **(n = 72)**	**NGO** **(n = 104)**	

**BASIC**						
Catchment area population	264,646	296,768	0.858	26,374	29,653	0.507
**STRUCTURE**						
Infrastructure and equipment						
*Physical infrastructure(# of amenities)*	4.56	4.87	0.103	3.37	3.78	0.076
*Examination room (# of items present)*	7.32	7.57	0.406	6.68	7.06	0.099
Management						
*System for review of management (%)*	91.5	92.5	0.342	82.2	69.6	0.010
*System for collecting client opinion (%)*	74.3	78.2	0.555	58.4	65.9	0.203
*Routine quality assurance program (%)*	62.5	72.0	0.154	44.1	49.7	0.779
*Last supervisory visit within 6 mths (%)*	91.2	80.4	0.147	95.6	92.6	0.022
*Facility has stock inventory and stock is organized and protected (%)*	79.6	53.4	0.000	57.6	29.1	0.000
*Stock inventory, quality (%)*	60.5	40.9	0.007	49.41	24.0	0.008
Availability of services						
*Number of days FP services provided*	5.1	5.2	0.342	5.1	5.5	0.043
*Trained provider always present (%)*	93.7	100.0	0.059	37.2	56.6	0.018
*# of FP methods offered (out of 14)*	6.8	5.8	0.026	4.9	3.9	0.001
*# of other reproductive health services offered (out of 6)*	4.4	4.2	0.296	3.6	3.5	0.301
Counseling						
*# of protocols on FP counseling(out of 5)*	1.0	0.8	0.310	1.1	0.9	0.179
*# of visual aids for demonstrating use of FP (out of 9)*	3.0	2.1	0.001	2.4	1.8	0.000
*Facility has private room for FP counseling (%)*	75.8	81.0	0.753	75.8	81.0	0.725
*Whether there is an individual client card for FP (%)*	92.0	59.1	0.000	74.4	49.4	0.037
*Number of years of FP experience of providers*	6.3	5.6	0.026	8.1	7.5	0.306
**PROCESS**						
*Waiting time*^1 ^*(minutes)*	69.2	67.8	0.954	65.2	21.9	*0.000*
Interpersonal (N = )	346	67		130	85	
*Privacy ensured during examination (%)*	79.3	73.0	0.039	81.1	84.7	0.004
*Asked clients about concerns with methods or currently used method (%)*	74.9	70.5	0.937	61.0	90.2	0.003
*Confidentiality assured (%)*	53.4	51.9	0.893	35.7	52.7	0.004
*Provider explained method use (%)*	73.0	79.0	0.965	72.0	64.3	0.273
*Provider prescribed injectable (%)*						
Technical						
*Reproductive history (out of 11)*	3.0	2.1	0.008	2.3	2.7	0.322
*Physical examination (out of 5)*	3.2	3.2	0.827	2.9	3.0	0.618
*Injectable procedure*^2 ^*(out of 9)*	3.8	3.8	0.971	3.6	3.9	0.137
*Duration of consultation (minutes)*	16.2	15.7	0.796	13.8	18.5	0.106

**Table 7:** Differences in attributes of quality (bivariate analysis) - Ghana

	Hospitals			Health Centers, Clinics & Other Facilities		
	**Mean** **Value**		**Signific. Level** **p-value**	**Mean** **Value**		**P**

	**Public** **(n = 42)**	**NGO** **(n = 12)**		**Public** **(n = 216)**	**NGO** **(n = 116)**	

**BASIC**						
Catchment area population	64,751	132,784	0.297	23,213	25,286	0.432
**STRUCTURE**						
Infrastructure and equipment						
*Physical infrastructure(# of amenities)*	4.8	4.7	0.471	3.4	4.2	0.000
*Examination room (# of items present)*	8.2	4.9	0.000	5.8	7.4	0.000
Management						
*System for review of management (%)*	98.0	100.0	0.590	65.5	39.8	0.000
*System for collecting client opinion (%)*	83.1	85.8	0.470	49.9	58.7	0.027
*Routine quality assurance program (%)*	73.0	49.8	0.389	21.2	8.2	0.002
*Last supervisory visit within 6 mths (%)*	88.8	83.1	0.260	76.9	58.2	0.002
Availability of services						
*Number of days FP services provided*	5.7	4.8	*0.018*	6.1	6.4	0.048
*Trained provider always present (%)*	98.0	100.0	0.590	37.4	53.6	0.006
*# of FP methods offered (out of 14)*	10.5	5.7	0.000	6.5	6.4	0.836
*# of other reproductive health services offered (out of 6)*	5.5	4.8	0.035	4.0	4.2	0.268
Counseling						
*# of protocols on FP counseling(out of 5)*	2.4	1.1	0.002	1.2	2.4	0.000
*# of visual aids for demonstrating use of FP (out of 9)*	5.0	3.8	0.081	3.7	3.9	0.397
*Facility has private room for FP counseling (%)*	77.4	78.7	0.600	76.8	84.4	0.119
*Whether there is an individual client card for FP (%)*	100.0	76.9	0.001	90.5	82.6	0.072
*Number of years of FP experience of providers*						
**PROCESS**						
*Waiting time*^1 ^*(minutes)*	30.8	38.0	0.612	24.5	33.2	0.149
Interpersonal						
*Privacy ensured during examination (%)*	73.8	71.6	0.096	83.1	90.5	0.008
*Asked clients about concerns with methods or currently used method (%)*	78.3	84.9	0.270	73.5	83.4	0.089
*Confidentiality assured (%)*	37.0	40.8	0.355	46.5	36.1	0.311
*Provider explained method use (%)*	70.1	70.1	0.856	75.7	73.3	0.248
*Provider prescribed injectable (%)*	68.3	68.8	0.761	71.9	81.1	0.555
Technical						
*Reproductive history (out of 11)*	3.0	2.6	0.438	2.2	2.2	0.822
*Physical examination (out of 5)*	2.4	2.4	0.883	2.2	2.2	0.529
*Injectable procedure*^2 ^*(out of 9)*	6.6	6.3	0.337	6.1	6.6	0.007
*Duration of consultation (minutes)*	28.3	24.1	0.466	25.9	22.8	0.251

#### Structural attributes of quality

In general, there did not appear to be systematic differences in infrastructure and equipment at the hospital level, with the exception of hospitals in Ghana. At the health center level and below, private facilities in all three countries scored higher on measures of physical infrastructure and necessary equipment in examination rooms.

On the other hand, public facilities - both hospitals and lower - tended to offer more FP methods than private facilities. Public Ghanian hospitals offered 10.5 FP methods on average, considerably more than private Ghanian hospitals which offered 5.7 methods on average. No statistically significant differences in FP availability were apparent at lower level facilities. Further, public facilities fairly consistently had higher levels of FP guidelines and protocols available, had more visual aids, and were more likely to have individual client cards than private facilities.

Only in Tanzania were measures of management systems significantly better at both public hospitals and health centers relative to private facilities. For example, nearly 80% of public hospitals in Tanzania had a stock inventory that was organized and protected as compared with only 60% of private/NGO hospitals. Similarly, 64% of public health centers had similar stock inventory systems as compared with less than half of private facilities.

#### Process attributes

While the picture surrounding structural quality at public and private facilities was mixed, process quality was clearly better at private facilities. In no country and at neither hospitals nor health centers were process measures of quality statistically significantly better at public relative to private facilities. For example, over 90% of clients at private health centers in Kenya reported that providers asked about client concerns regarding methods or method use as compared to only 61% of providers at public health centers. The probability that confidentiality would be assured also appeared higher at private relative to public facilities.

Further, waiting times were nearly always considerably longer at public facilities than private facilities, at least at lower level facilities. In both Tanzania and Kenya, FP clients waited over 40 minutes longer on average at public sector health centers than at private health centers and clinics. No statistically significant differences in waiting times were found at hospitals in any of the three countries; the duration of the FP consultation was roughly the same across public and providers in all countries as well.

There appeared to be few differences in technical aspects of quality between private and public facilities. In Kenya, providers in public hospitals were more likely to take reproductive histories but no such difference appeared in hospitals in the other two countries. At lower level facilities, private providers performed better in Tanzania, but not in the other two countries. Physical exams also appeared to be similar, as were injectable procedures.

### Differences in satisfaction: bivariate analysis

At all levels and in all three countries, respondents reported higher satisfaction with the quality of the examination and treatment at private facilities (Table 8, Table 9, Table 10). In some cases, these differences were not large though they were statistically significant. For example, in Tanzania 96.8% of respondents reported "no problem" with the quality of treatment in public hospitals versus 99.4% of respondents at private hospitals. While this difference appears small, it was statistically significant at the 5% level. Differentials in perceptions of quality appeared largest with waiting times. For example, roughly 40% of clients reported problems with waiting times at public clinics in Kenya versus only 5% of clients at private clinics.

**Table 8:** Differences in Ratings of Satisfaction (Percent saying "No problem"), Tanzania

	Hospitals			Health Centers, Clinics, & Other Facilities		
	**Mean** **Value**		**Signific. Level** **p-value**	**Mean** **Value**		**Signific**. **Level** **p-value**

	**Public** **(n = 87)**	**NGO** **(n = 24)**		**Public** **(n = 314)**	**NGO** **(n = 55)**	

**PROBLEMS**						
Time you waited	69.8	70.4	0.914	74.1	85.8	0.062
Ability to discuss problems or concerns about your health with the provider	94.8	98.8	0.022	96.7	100.0	0.002
Amount of explanation you received about any problem or method of FP	94.8	98.8	0.011	95.5	94.8	0.846
Quality of the examination and treatment provided	96.8	99.4	0.023	95.9	100.0	0.000
Privacy from having others see the examination	94.8	90.2	0.586	96.0	92.7	0.630
Privacy from having others hear your consultation discussion	95.2	100.0	0.001	95.9	90.1	0.391
Availability of medicines or methods at this facility	83.6	92.2	0.095	79.5	94.9	0.000
Hours of service at this facility	91.6	87.9	0.432	88.7	97.6	0.002
Number of days services are available to you	94.9	85.3	0.210	92.1	92.4	0.932
Cleanliness of the facility	87.0	94.0	0.131	87.0	97.4	0.003
How the staff treated you	93.8	99.4	0.000	92.4	100.0	0.000
Cost for services or treatment	93.8	95.1	0.762	96.0	92.4	0.395
Total "yes"	10.9	11.1	0.389	10.9	11.4	0.045
						
**OUTCOME**						
*Client satisfaction (%)*	51.5	45.6	0.608	46.9	70.8	0.016

**Table 9:** Differences in Ratings of Satisfaction (Percent saying "No problem") -Ghana

	Hospitals			Health Centers, Clinics, & Other Facilities		
	**Mean** **Value**		**Signific. Level** **p-value**	**Mean** **Value**		**Signific**. **Level** **p-value**

	**Public** **(n = 172)**	**NGO** **(n = 32)**		**Public** **(n = 242)**	**NGO** **(n = 165)**	

**PROBLEMS**						
Time you waited	90.6	96.0	0.220	90.1	93.1	0.300
Ability to discuss problems or concerns about your health with the provider	97.2	100.0	0.046	93.9	97.0	0.245
Amount of explanation you received about any problem or method of FP	96.3	100.0	0.008	92.0	96.1	0.172
Quality of the examination and treatment provided	96.2	96.5	0.952	93.2	97.1	0.122
Privacy from having others see the examination	97.1	100.0	0.047	95.5	95.7	0.926
Privacy from having others hear your consultation discussion	96.7	100.0	0.046	94.8	96.5	0.469
Availability of medicines or methods at this facility	94.9	98.2	0.208	96.4	97.6	0.537
Hours of service at this facility	94.8	96.0	0.762	93.0	97.7	0.032
Cleanliness of the facility	96.3	92.5	0.394	88.7	94.0	0.168
How the staff treated you	97.9	100.0	0.096	96.4	98.4	0.280
Other	88.8	89.6	0.928	84.3	96.1	0.009
						
Total "yes"	10.5	10.7	0.194	10.2	10.6	0.046
						
**OUTCOME**						
*Client satisfaction (%)*	71.1	76.3	0.341	59.2	81.2	0.000

**Table 10:** Differences in Ratings of Satisfaction (Percent saying "No problem") - Kenya

	Hospitals			Health Centers, Clinics, & Other Facilities		
	**Mean** **Value**		**Signific. Level** **p-value**	**Mean** **Value**		**Signific**. **Level** **p-value**

	**Public** **(n = 346)**	**NGO** **(n = 67)**		**Public** **(n = 130)**	**NGO** **(n = 85)**	

**PROBLEMS**						
Time you waited	59.4	69.0	0.262	60.3	95.2	0.000
Ability to discuss problems or concerns about your health with the provider	86.5	83.4	0.605	89.9	94.8	0.256
Amount of explanation you received about any problem or method of FP	8.83	88.4	0.991	85.9	93.2	0.119
Quality of the examination and treatment provided	88.3	93.9	0.196	89.0	99.0	0.001
Privacy from having others see the examination	90.5	82.8	0.238	87.6	87.2	0.961
Privacy from having others hear your consultation discussion	88.5	83.4	0.444	87.6	93.8	0.364
Availability of medicines or methods at this facility	73.8	82.8	0.108	67.6	90.6	0.014
Hours of service at this facility	83.1	88.0	0.294	86.8	99.0	0.001
Number of days services are available to you	88.0	90.4	0.532	89.2	98.1	0.009
Cleanliness of the facility	84.6	93.6	0.042	89.4	99.5	0.006
How the staff treated you	87.1	93.6	0.131	90.0	99.7	0.001
Cost for services or treatment	93.8	84.0	0.404	90.5	96.9	0.133
Total "yes"	10.1	10.3	0.669	10.1	11.5	0.000
						
**OUTCOME**						
*Client satisfaction (%)*	34.1	51.7	0.000	29.1	63.6	0.000

A second area of clear differences between public and private facilities was with the availability of medicines or contraceptive methods. For example, only two-thirds of respondents reported "no problem" with availability at public clinics in Kenya, versus 91% at private clinics. A similar result was found in Tanzania though not in Ghana. Perceptions of quality were high at both public and private facilities in Ghana. The highest levels of dissatisfaction were with the cleanliness of public health centers, for which 12% of respondents reported a problem.

**Figure 1:**
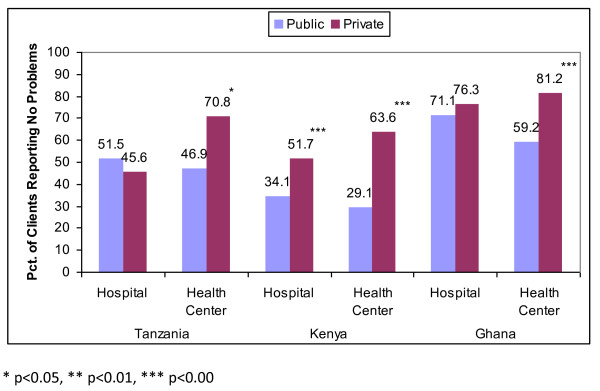
**Client satisfaction by Facility Management, Level and Country**.

Using the discrete measure of quality - the absence of any problems during an FP consultation - the differences were starker, as shown by Figure 1. In four out of six cases, satisfaction was higher at private facilities relative to public facilities. In Kenya, nearly two-thirds of FP clients at private health centers reported no problem as compared with just under one-third of FP clients at public health centers. There tended to be greater parity in satisfaction at hospitals relative to health centers, and in fact satisfaction at public hospitals was higher in Tanzania - but not at a statistically significant level - than at private hospitals, though in both cases only about half of clients reported no problems.

### Correlates of client satisfaction - multivariate regression analysis

We examined the correlates of client satisfaction among clients of private and public sector facilities in each of the countries. Overall, even with controls for specific attributes of quality, private facilities seem to have higher levels of client satisfaction than public facilities (Table 11). This was true at the clinic level in all three countries and at the hospital level in Ghana. Further, the measures of quality that most impacted upon client perceptions of quality were those that were most directly observable by them, namely process attributes of quality, for which private facilities tended to score better.

**Table 11:** Factors Associated with Client Satisfaction (multivariate analysis) (Coefficient and standard error)

	Ghana				Kenya				Tanzania			
	Hospital		Clinic		Hospital		Clinic		Hospital		Clinic	
	
Independent Variables	No problems	Index of satisfaction	No problems	Index of satisfaction	No problems	Index of satisfaction	No problems	Index of satisfaction	No problems	Index of satisfaction	No problems	Index of satisfaction
FACILITY CHARACTERISTICS												
NGO	0.4178	0.3034*	0.7329**	0.2128	0.4955	0.2300	0.4952	0.6930***	-0.4634	0.0566	2.4378*	1.1462*
	0.231	0.014	0.002	0.149	0.178	0.099	0.119	0.000	0.108	0.760	0.029	0.014
Urban					0.027	0.0382	-0.8547	-0.8163*				
					0.967	0.91	0.19	0.013				
Log (catchment pop)	0.4741	0.1487	0.1633	-0.1145	-0.0402	-0.0439	-0.0101	-0.0563	0.0933	-0.0037	-0.1155	-0.0689
	0.051	0.194	0.534	0.369	0.675	0.48	0.952	0.561	0.268	0.95	0.265	0.278
STRUCTURE												
Facility inventory	-0.0166	-0.0337	0.0993	0.0345	-0.0956	-0.0549	0.1234	0.1243**	0.1091*	0.0628*	-0.0587	-0.0129
	0.865	0.429	0.053	0.199	0.117	0.117	0.125	0.005	0.022	0.048	0.11	0.577
Trained provider present 24 hours		-0.219	-0.0696	-0.0488	0.2186	-0.1617	0.7691*	0.0377	0.034	0.176	0.1819	0.1041
		0.262	0.703	0.703	0.518	0.548	0.038	0.841	0.967	0.74	0.309	0.373
Supervisory visit in last 6 months	-1.1562*	-0.3568	-0.1475	-0.0381	-0.3477	-0.3453*	-1.4670*	-0.1202				
	0.028	0.057	0.580	0.789	0.260	0.033	0.042	0.736				
Number of staff					0.0018	0.0011	0.0015	0.0018	-0.0004	0.0001	0.0171	0.0077
					0.070	0.179	0.611	0.18	0.742	0.911	0.178	0.213
Number of days FP offered	0.4559*	-0.0724	0.0512	-0.0475	0.0479	-0.0531	0.0267	-0.1272	-0.4142	0.0081	0.0953	0.0085
	0.049	0.629	0.380	0.126	0.841	0.606	0.885	0.219	0.124	0.957	0.295	0.886
System of quality assurance	-0.0946	0.0541	0.0457	-0.0835	-0.049	0.0566	0.0415	-0.1356	0.0086	0.3349	0.1257	0.0177
	0.751	0.696	0.834	0.517	0.83	0.700	0.874	0.331	0.979	0.225	0.424	0.84
Total FP methods offered	0.016	0.0255	-0.0587	-0.0011	-0.0413	-0.0839*	-0.2152	0.0606	0.0781	0.0239	0.095	0.1195
	0.861	0.486	0.289	0.977	0.478	0.023	0.079	0.294	0.248	0.582	0.222	0.085
Protocols on FP followed	-0.069	0.0495	-0.0117	-0.0297	0.0839	0.1239	-0.2139	-0.0046	0.0531	0.0086	0.1396	0.1376**
	0.563	0.489	0.885	0.623	0.418	0.067	0.154	0.955	0.641	0.900	0.055	0.001
FP client record maintained		0.0612	0.2400	0.0341	-0.2400	-0.3421*	1.1700**	0.3688	0.1208	-0.0803	-0.1319	-0.1831
		0.936	0.511	0.831	0.455	0.022	0.002	0.064	0.756	0.619	0.611	0.187
Quality stock inventory	0.4317*	-0.0026	-0.0481	0.121	-0.0622	-0.0477	0.2252	0.1147	0.0298	0.0753	0.0288	-0.0095
	0.014	0.970	0.655	0.115	0.800	0.716	0.447	0.572	0.88	0.656	0.862	0.926
Number trained					-0.0589	-0.1385**	0.2183	-0.0317				
					0.352	0.004	0.099	0.648				
PROCESS												
Visual & auditory privacy ensured	0.0516	-0.1216	-0.176	0.0373	-0.1143	-0.0926	0.3986	0.0989	-0.0104	0.3347	0.0693	0.1567
	0.891	0.603	0.437	0.824	0.61	0.626	0.286	0.656	0.984	0.433	0.727	0.255
No. of repro health and phys exam	0.0279	0.013	0.0203	0.0308*	0.0273	0.0431*	0.1418**	0.0268	-0.0251	-0.0227	0.0133	0.0222
elements performed	0.310	0.23	0.366	0.05	0.247	0.012	0.003	0.307	0.352	0.234	0.565	0.117
Client concerns noted	0.4387	0.0519	-0.0547	-0.1181	-0.0791	0.0142	0.2557	0.0177	-0.1051	-0.1891	-0.2422	-0.0059
	0.082	0.604	0.764	0.369	0.716	0.917	0.511	0.932	0.647	0.166	0.175	0.961
Confidentiality assured	-0.0883	0.1231	0.0516	-0.1013	0.373	0.4389**	0.4255	-0.0138	0.4644	0.2149	-0.1702	-0.071
	0.773	0.385	0.785	0.466	0.063	0.002	0.133	0.926	0.094	0.139	0.310	0.505
Client told about side effects	-0.0864	0.0104	0.442	0.5430**	-0.1149	-0.072	-0.2877	-0.0759				
	0.773	0.945	0.055	0.005	0.512	0.48	0.502	0.71				
Injectable method prescribed	0.1259	0.149	0.2749	0.3884*	0.0921	-0.113	0.0998	-0.2611	0.5246**	0.3512*	0.0821	0.1483
	0.618	0.264	0.135	0.032	0.668	0.271	0.785	0.174	0.002	0.036	0.560	0.142
Waiting time	-0.0048*	-0.0021*	-0.009***	-0.0037**	-0.008***	-0.004***	-0.011***	-0.003	-0.007***	-0.0030***	0.0406	0.0237**
	0.019	0.042	0.000	0.001	0.000	0.000	0.000	0.090	0.000	0.000	0.096	0.008
CLIENT CHARACTERISTICS												
Age	0.0003	0.0134	0.0029	0.0036	0.0027	-0.0058	-0.0188	-0.0219	-0.0358	-0.0002	-0.0048	-0.0019
	0.988	0.294	0.788	0.446	0.839	0.468	0.402	0.159	0.074	0.987	0.604	0.732
Primary school educ	0.5967*	0.0369	-0.1207	0.3034*	-0.1155	-0.0878	-0.2297	-0.1586	-0.0238	-0.0342	-0.0602	0.0201
	0.019	0.798	0.563	0.033	0.293	0.186	0.263	0.237	0.820	0.695	0.463	0.726
Secondary school educ.	0.8252**	0.0824	-0.1366	0.2054								
	0.002	0.587	0.380	0.086								
Intercept	-4.5372*	-0.3599	-1.6069	-0.7146	1.8286	3.1225***	-0.675	0.5066	0.2395	-1.4728	-2.2200	-1.9172
	0.042	0.597	0.204	0.260	0.288	0.001	0.755	0.710	0.913	0.314	0.272	0.059

N	197	204	407	407	390	390	208	208	322	322	450	450
r2		0.1158		0.1735		0.2579		0.2372		0.1653		0.0825
F	2.1151	.	2.879	1.2948	6.3419	14.0259	3.9912	2.7142	2.1756	4.4956	1.3143	1.1052

* p < 0.05, ** p < 0.01, *** p < 0.001

#### Structure

Few measures of structural quality appeared to affect client satisfaction. Service availability - as measured by the number of FP methods offered and the number of days per week that FP services were offered - had little impact on client satisfaction. Whereas public facilities appeared in bivariate analyses to have better management systems (e.g. having a system of Quality Assurance, having appropriate stock management procedures in place) - perhaps because competitive mechanisms and for-profit motives that encourage accountability at private facilities are less prevalent at public facilities - these did not show a statistically significant association with client satisfaction in the multivariate analyses. Having a supervisory visit in the last 6 months was actually negatively associated with client satisfaction in two cases, perhaps because more troubled facilities are likely to require closer supervision. Other structural factors that had no influence were the presence of FP protocols and guidelines, training of staff, and number of staff.

#### Process

Consistently, longer waiting times were negatively associated with client satisfaction at all facilities and in all countries. Conversely, the performance of more physical and reproductive health exam elements increased satisfaction, as did prescribing an injectable method. Each of these aspects of quality are easily discernible, even to an untrained client, and therefore likely to perceptibly influence satisfaction, though they may have little impact on meeting the overall FP needs of clients. Other process factors had little influence, including the maintenance of confidentiality, informing clients of potential side effects, and noting client concerns.

## Discussion

This study has focused on measuring the extent of quality differentials between public and private FP providers in three countries and then relating client satisfaction to both clients' perceptions and experts' assessments of the quality of FP services. As expected we found significant quality differences between public and private providers, mostly at lower level facilities, which accords with economic theory regarding supply side responsiveness to client demand. On the other hand, we found little evidence that private providers skimped on less (client) perceptible technical measures of quality.

We found little evidence that client satisfaction bears much relationship with technical aspects of quality, as perceptions of adherence to appropriate family planning procedures require greater technical knowledge and awareness than is likely to be possessed by the typical FP client. This is consistent with previous research [8,21]. As noted by one set of researchers, "Consumers are usually unable to assess the technical quality of services, with the result that they place more weight on aspects of perceived quality, such as the interpersonal skills of providers and the comfort of the environment in which treatment occurs, both of which may be unrelated to technical competence. They may, therefore, be more exposed to inadequately qualified practitioners providing care of very poor quality" ([21] p. 326). Previous studies [37,38] have found that the quality of client-provider interactions contributes significantly to client satisfaction and contraceptive continuation. In this study - and in confirmation with economic incentives - these aspects also tended to be better at private and NGO facilities relative to public facilities, at least at the clinic level.

These results, however, do not imply that client satisfaction should be the principal goal of providers. In fact, client satisfaction is inextricably linked to expectations, which may differ across clients of different types of facilities. Certainly the evidence exists to show that higher levels of client satisfaction with process measures of quality increases the likelihood of contraceptive use and continuation [10]. But structural measures of quality - such as frequent shortages of methods or inappropriate guidance - are also likely to inhibit long-term contraceptive continuation. Ensuring that clients are appropriately informed about methods, their uses, side effects and limitations; are correctly given physical exams; and are seen by trained providers are all important determinants of quality and contraceptive use [1,2]. Other convenience measures, such as waiting times, seem to be important determinants of client satisfaction, but are less likely to have any impact upon the technical quality of services, though they may impact longer term use of methods if they inhibit clients from returning for follow-up visits. Regardless, FP providers would obviously be well-advised - regardless of their incentive structures - to monitor and ensure all aspects of FP quality.

One shortcoming of this analysis was the inability to distinguish between for-profit and not-for-profit private facilities, a lament shared by previous researchers [11]. This represents an important limitation because the incentive structures - such as the trade-off between cost savings and quality - may differ considerably between the two types. Nonetheless, in at least two of the countries, the size of the nongovernmental mission sector - and therefore as a proportion of our facility sample - is not large. In Kenya, for example, the private medical sector is the predominant private family planning provider, constituting 80% of all private provision of family planning. Similarly, in Ghana, the nonprofit nongovernmental sector provides only a very small percentage of overall family planning supply [29]. As a result, the results for these two countries are more likely a reflection of differences between government providers relative to private for-profit providers, rather than religious and mission providers.

An additional limitation relates to the use of exit interviews as a source of client satisfaction. Exit interviews by definition involve a sample of clients who have already made a choice to appear at a specific facility and are therefore likely to believe that the facility will be minimally satisfactory. Non-clients may have chosen to go elsewhere or to do nothing, simply because they do not believe that quality at a particular facility will be satisfactory. For example, potential clients who are particularly intolerant of long waiting times may eschew public facilities specifically because of perceptions that waiting times will be unacceptable. Therefore, the sample of actual clients - and those completing the exit interviews - may represent a group who cares less about waiting times, thereby understating the true effect of waiting times on client satisfaction. To fully address the effects of quality on client satisfaction would require a random sample of the larger population of reproductive age women, linking their reproductive health choices - and satisfaction - to the supply environment as measured with a SPA.

## Conclusions

This study makes an important contribution by highlighting differences in quality between public and private facilities according to three aspects of quality and fills a gap in knowledge on this topic by linking structural and process quality to client satisfaction. The finding of significantly lower technical quality at lower level public facilities should raise some concern. Further study is clearly warranted to determine the principal causes of quality deficiencies - insufficient training of personnel, resource shortages, limited management oversight or some other reason. Our findings hint at a role for each of these causes.

Referring to one of the limitations of this analysis, future large-scale studies, such as the SPAs, should make a point to distinguish between different types of private facilities, and to make this data available to researchers. Additionally, the value of SPAs could be further enhanced if they were timed and coordinated to cover the same populations and catchment areas covered by large scale population surveys such as the DHS. Such a mechanism, as noted above, could provide a richer means by which to evaluate the effects of the health service supply environment on a wide range of health behaviors and choices in developing country populations.

Finally, as the private sector appears to be an important provider of reproductive health services in the three countries studied, care should be taken to prevent the implementation of policies or regulations that significantly burden or hamper the functioning of the private sector lest national-level reproductive health indicators suffer as a result.

## Competing interests

The authors declare that they have no competing interests.

## Authors' contributions

All authors participated in research design and conceptualization, data analysis, writing, and revision of this manuscript. All authors have read and approved the final manuscript.

## Authors' information

Paul Hutchinson, PhD, and Mai Do, DrPH, MD, are Assistant Professors in the Department of International Health and Development in the Tulane University School of Public Health and Tropical Medicine. Sohail Agha, PhD, is a Senior Technical Advisor with Population Services International (PSI).

## Pre-publication history

The pre-publication history for this paper can be accessed here:

http://www.biomedcentral.com/1472-6963/11/203/prepub
